# Birth Weight, Current Anthropometric Markers, and High Sensitivity C-Reactive Protein in Brazilian School Children

**DOI:** 10.1155/2015/846376

**Published:** 2015-03-19

**Authors:** Camile Boscaini, Lucia Campos Pellanda

**Affiliations:** ^1^Post-Graduation Program in Health Sciences—Cardiology, Instituto de Cardiologia/Fundação Universitária de Cardiologia, Porto Alegre, RS, Brazil; ^2^Federal University of Health Sciences of Porto Alegre, Porto Alegre, RS, Brazil; ^3^Research Unit, Avenida Princesa Isabel 370, Santana, 90620-000 Porto Alegre, RS, Brazil

## Abstract

Studies have shown associations of birth weight with increased concentrations of high sensitivity C-reactive protein. This study assessed the relationship between birth weight, anthropometric and metabolic parameters during childhood, and high sensitivity C-reactive protein. A total of 612 Brazilian school children aged 5–13 years were included in the study. High sensitivity C-reactive protein was measured by particle-enhanced immunonephelometry. Nutritional status was assessed by body mass index, waist circumference, and skinfolds. Total cholesterol and fractions, triglycerides, and glucose were measured by enzymatic methods. Insulin sensitivity was determined by the homeostasis model assessment method. Statistical analysis included chi-square test, General Linear Model, and General Linear Model for Gamma Distribution. Body mass index, waist circumference, and skinfolds were directly associated with birth weight (*P* < 0.001, *P* = 0.001, and *P* = 0.015, resp.). Large for gestational age children showed higher high sensitivity C-reactive protein levels (*P* < 0.001) than small for gestational age. High birth weight is associated with higher levels of high sensitivity C-reactive protein, body mass index, waist circumference, and skinfolds. Large for gestational age altered high sensitivity C-reactive protein and promoted additional risk factor for atherosclerosis in these school children, independent of current nutritional status.

## 1. Introduction

Environmental factors acting early in life influence the risk of developing adult cardiovascular disease (CVD). Birth weight and/or infant weight and accelerated weight gain during childhood are associated with an increased risk of these disorders [1]. Recent research has focused on the role of chronic, low-grade inflammatory processes in the pathophysiology of a wide range of chronic degenerative diseases [2, 3]. In particular, elevated concentrations of C-reactive protein (CRP) have been consistently associated with increased risk for cardiovascular disease [4]. Serum CRP levels were a long-term predictor of risk of cardiovascular and noncardiovascular mortality independent of traditional risk factors or other inflammatory markers, such as fibrinogen and leukocyte count [5].

Thus, both high sensitivity C-reactive protein (hs-CRP) and low birth weight have emerged as predictors of CVDs, but the relationship between these two variables is still unclear. Thinness at birth or during infancy and accelerated body mass index (BMI) gain during childhood/adolescence are associated with a proinflammatory/prothrombotic state in adult life. An altered inflammatory state could be one link between small newborn/infant size and adult cardiovascular disease [6].

Study has shown that low birth weight is associated with higher concentrations of hs-CRP in adults [6], but studies in children failed to demonstrate this association. Considering that metabolic changes associated with hs-CRP concentrations increase from childhood to adulthood, cumulative effects along the life course may explain these differences [7].

Therefore, the objective of this study was to further investigate the relationship between hs-CRP and birth weight and other risk factors (obesity, dyslipidemia) for cardiovascular disease in school children in southern Brazil.

## 2. Patients and Methods

### 2.1. Design and Location of Study

We conducted a population-based cross-sectional study in the city of Garibaldi, south Brazil. The city has currently 30,165 inhabitants and 21 elementary public schools with a total of 1,464 students 5–10 years old. Data was collected between 2011 and 2012, after approval of the Research Ethics Committee of the Institute of Cardiology of RS. All parents signed an informed consent and all children agreed to participate.

### 2.2. Population

Sample size was determined according to the study by Rondó et al. (2013) [7], which identified altered hs-CRP levels in 27.7% of Brazilian children aged 5 to 8 years. Considering the total number of students, cluster sampling (clusters = schools), a 95% confidence level, and a 5% error margin, it would be necessary to study 481 children. Exclusion factors were use of medications that could interfere with laboratories results, current infectious diseases or fever, and history.

### 2.3. Current Anthropometric Parameters

Anthropometric measurements were repeated three times, nonconsecutively, and mean values were used in the analyses. Participants should be barefoot and wearing light clothes (shorts for boys and shorts and t-shirts for girls). Weight was measured using a digital scale (Techline) with a variation of 100 g. For height measurements, children stood in vertical position, with feet parallel and with the heels, shoulders, and buttocks touching the wall, and a stadiometer with accuracy of 0.1 cm was used.

BMI, determined as weight in kilograms divided by height in meters squared, was used to assess the nutritional status, based on the BMI-for-age standards determined by the World Health Organization (WHO) [8] and values ≥+1 were considered overweight [8].

For measurement of the waist circumference (WC), children were placed in standing position, with the abdomen relaxed and arms along the body. The measuring tape was positioned around the natural waist line, in the narrower region between the thorax and the pelvis, at the midpoint between the last rib and the iliac crest, with a firm but not compressive force. The measurement was made at the time of expiration [9]. Body composition was assessed through the sum of the tricipital and subscapular skinfolds.

All measures were performed by a registered dietitian (CB) and two previously trained undergraduate nutrition students.

### 2.4. Data Related to Child Birth

Parents or caretakers were asked to bring birth registrations on the day of the scheduled interview, for collection of information on birth weight and gestational age at birth. Birth weight was categorized as proposed by the WHO [8], with the following cut-off points: low weight (<2,500 g), insufficient weight (2,500–3,000 g), and adequate weight (3,000–4,000 g).

Birth weight according to gestational age was classified according to the curve developed by Lubchenco et al. (1963) [10]: small for gestational age (SGA) when below the 10th percentile, appropriate for gestational age (AGA) when between the 10th and 90th percentile, and large for gestational age (LGA) when above the 90th percentile.

### 2.5. Biochemical Parameters

Blood was collected according to the protocol provided by the Brazilian Society of Cardiology [11] and was conducted by a biochemist, during the morning and after the appropriate fasting period (8–12 hs). All children were necessarily accompanied by parents or guardians. About 6 mL of blood were collected by venipuncture in the cubital fossa, using disposable material. The blood samples were stored at −20°C in heparinized vacutainer tubes. Serum levels of total cholesterol (CT), triglycerides (TG), and glucose were determined with an automated enzymatic method. An automated homogeneous assay was used for determination of high density lipoprotein (HDL-c) levels, and insulin was evaluated by chemiluminescence. The low density lipoprotein (LDL-c) level was calculated by the Friedewald formula [12]. hs-C-reactive protein levels were studied by nephelometric high-sensitivity assay. The homeostasis model assessment method for insulin resistance (HOMA-IR) was used for the evaluation of insulin resistance, by multiplying the glycemic index (mmol/L) by the insulin index (*μ*UI/mL), both measured in fasting, and dividing by 22.5 [13].

### 2.6. Statistical Analyses

The statistical analyses were performed with the Statistical Package for the Social Sciences (SPSS), version 21.0. The chi-square test was used for categorical variables. All data sets were tested for normality with Kolmogorov Smirnov Test. Variables with normal distribution were analyzed with one-way ANOVA, and asymmetric variables were analyzed with Mann-Whitney test. For regression analyses with control of confounding factors, linear models were used for data with normal distribution, and linear models with gamma distribution were used for asymmetric variables. The best adjusted model between all anthropometric variables was verified by the Akaike information criteria. Statistical significance was set at alpha <0.05.

## 3. Results

A total of 612 school children were evaluated. Of those, 572 presented complete data and were included in this study. Mean age of the participants was 8.6 (±1.46) years, and 51.5% were male. Analysis of maternal age at birth showed that 16.7% of the school children were born to teenage mothers, and 86.1% were Caucasian.

The prevalence of school children with very low and low birth weight was 7.7% and 5.6%, respectively, and 86.8% had adequate birth weight. The frequency of school children born SGA was 2,1%, AGA 79.6% and LGA was 18.2%.

A higher proportion of boys than girls (23.7% versus 12.5%) had LGA (*P* < 0.001). The maternal age and ethnicity did not differ significantly with the hs-CRP (Table 1). The BMI showed higher values of z-score in school children with LGA (*P* < 0.001). The same was observed for the waist circumference and skinfolds (*P* < 0.001 and *P* = 0.02, resp.) (Table 2).

School children with LGA showed higher values of hs-CRP, HOMA-IR, and insulin, in comparison with SGA (*P* < 0.001, *P* = 0.03, and *P* = 0.02, resp.). Other biochemical parameters analyzed showed no statistically significant differences (Table 2).

The association between birth weight and the three anthropometric markers analysed, BMI, WC, and skinfolds, remained statistically significant in the regression model analysis (*P* < 0.001, *P* = 0.001, and  *P* = 0.015, resp.). The association between hs-CRP levels and LGA was also present after adjusting for gender, body mass index, waist circumference, and skinfolds (*P* < 0.001) (Table 3).

## 4. Discussion 

The results observed in the evaluation of school children in southern Brazil showed that high birth weight is associated with higher levels of high sensitivity C-reactive protein, body mass index, waist circumference, and skinfolds.

The prevalence of school children born with LGA was 18.2%, and the prevalence of school children born SGA was 2.1%. Scientific evidences around the world have shown concern with the increasing prevalence of low birth weight, which has been associated with several health complications. However, the present study highlights that high birth weight may also be related to several complications shown by anthropometric, metabolic, inflammatory, and biochemical results, thus contributing to the early development of the cardiovascular risk factors in school children. This represents a new association between birth weight and early outcomes associated with overweight, dyslipidemia, and metabolic and inflammatory changes. Singhal et al. [14] showed that high birth weight, determined by the increase of one standard deviation in the BMI, results in higher values of this anthropometric indicator in adolescence. High birth weight and rapid weight gain in the first 3 months of life contribute to elevating the BMI at 2 years of age, demonstrating the early interaction between high birth weight and childhood overweight [15].

However, the association between birth weight and BMI contradicts considerable evidence that a high birth weight programs less susceptibility rather than greater susceptibility to cardiovascular disease (CVD) risk factors [16, 17]. Previous studies have shown that babies who are born small and then show rapid catch-up growth have in a recent systematic review been shown to be more obese in later life [18]. Some authors consider that catch-up growth in the first few weeks of postnatal life is particularly disadvantageous [19, 20] whereas others suggest that low birth weight children who grow excessively in later childhood are also particularly at risk of later obesity [21]. The prevalence of low birth weight found in our study was similar to that mentioned in national data released by the United Nations [22]. The study “Prematurity and its possible causes” investigated data on low weight of more than 6,000 Brazilian children, concluding that the incidence of about 8% of low birth weight in total births has remained stable since 2000. However, a recent study on children born in 2013 in China showed a prevalence of 1.7% of low birth weight [15]. Ethnic differences, or age of the mother, may have influence on the different prevalence of cases of low birth weight in different countries. In Brazil, low birth weight is more frequent in black mothers (9.4%), followed by white (8.3%) and mulatto mothers (8.2%). The lowest rates were found between Asian and indigenous women: 7.6% and 7.7%, respectively [23]. In the present work, no significant relationship was observed between maternal age or ethnicity and birth weight classification according to gestational age. However, a higher proportion of boys than girls had LGA. In a recent study, no statistically significant difference was observed between the birth weight of boys and girls [15]. It should be noted that the method used for classification of birth weight considered only this variable, whereas in the present study the variable weight at birth was determined according to gestational age, classifying individuals by percentiles.

The analysis of birth weight and metabolic indicators showed higher values of glycaemia, insulin, and HOMA-IR in the children with LGA, but this association was not significant when BMI was considered a controlling factor. Factors related to obesity, such as the accumulation of abdominal fat and hyperinsulinemia, are also associated with the thrombogenic and inflammatory profile. Atherogenic, thrombogenic, and inflammatory metabolic changes contribute to a higher risk of coronary heart disease in obese children and adolescents, with accumulation of fat in the abdominal area [23]. It is known that overweight is an important cause of altered levels of insulin, blood sugar, and consequently HOMA-IR. Genetically determined insulin resistance could result in impaired insulin-mediated growth of fetal muscle, and the continuation of this pattern of body composition would lead to less muscle mass later in life [24].

High levels of inflammatory markers such as IL-6, tumor necrosis factor, and CRP are related to general and abdominal obesity. Children with overweight and obesity have higher concentrations of serum CRP, which supports the hypothesis of a relationship between childhood obesity and the presence of systemic inflammatory substances [25]. Inflammation has been understood to be a key pathogenic mechanism in the initiation and progression of cardiovascular disease (CVD) [26] and great attention has been given to inflammatory markers for their ability to predict CVD risk [3].

Our results showed that levels of hs-CRP in school children at school age are significantly higher in cases of LGA. However, in a cohort study [27], an inverse relationship between CRP and BMI values was found at 2, 11, and 21 years of age. Similarly, no statistically significant association between birth weight and hs-CRP was observed in a study in Brazil with children of 5–8 years of age [28].

Thinness at birth and/or in infancy is associated with higher fibrinogen, hs-CRP in adulthood. Both in-utero influences and greater adiposity due to BMI gain in childhood/adolescence could be implicated consolidating the need to prevent excessive BMI gain in childhood [6].

All anthropometric markers measured in this study (BMI, WC, and skinfolds) were increased in the participants who were LGA when compared to SGA. Similar results were found in the study of Rondó et al. [28], who described a positive association between WC and elevation of hs-CRP levels. Thomas et al. [29] observed a relationship between fatness and higher values of CRP, suggesting that a reduction of body fat can decrease the levels of CRP and thus prevent future cardiovascular events.

Infancy is understood as a critical period for the development of obesity for many reasons, but primarily because infants are experiencing food transitions, establishment of eating habits, and, too often, the early development of excess adiposity. Recently, more evidence has become available regarding the associations of early weight status and rapid growth with obesity and related problems in later life [30, 31]. For instance, Harrington's study showed that more than half of the overweight children aged 2 to 20 years became overweight before the age of two [31].

Body measures, specifically in the pediatric phase, change according to growth and development. The evaluation of the normality of these measures becomes complex but represents an important tool for evaluating the growth and nutritional status of children and adolescents. In this age group, nutritional changes usually reflect on growth, so that anthropometric parameters are important indicators for the assessment of nutritional status [32].

## 5. Conclusions

High birth weight and/or in infancy is associated with higher levels of high sensitivity C-reactive protein, BMI, WC, and skinfolds. Large for gestational age altered high sensitivity C-reactive protein and promoted additional risk factor for atherosclerosis in these school children, independent of current nutritional status.

## Figures and Tables

**Table 1:** Birth weight, maternal characteristics, and gender in Brazilian school children (*n* = 612), 2013.

	Total		SGA birth		AGA birth		LGA birth		*P* value
	*n*	%	*n*	%	*n*	%	*n*	%	
Maternal age at child birth									
<20 years	101	16.7	2	2.2	71	78.0	18	19.8	0.92
≥20 years	505	83.3	10	2.2	371	79.8	84	18.1	
Ethnicity									
Other ethnicities	85	13.9	0	0.0	57	79.2	15	20.8	0.35
White	527	86.1	12	2.5	388	79.7	87	17.9	
Gender									
Male	315	51.5	4	1.4	215	74.9	68	23.7	<0.001
Female	297	48.5	8	2.9	230	84.6	34	12.5	

SGA: small for gestational age.

AGA: appropriate for gestational age.

LGA: large for gestational age.

**Table 2:** Association between birth weight and anthropometric markers, lipid profile, glucose, HOMA-IR index, and hs-CRP in Brazilian school children (*n* = 612), 2013.

	SGA birth			AGA birth			LGA birth			*P* value
	*n*	Mean	SD	*n*	Mean	SD	*n*	Mean	SD	
Anthropometric parameters										
Body mass index (*z*-score)	12	−0.6	1.3	444	0.5	1.4	102	0.8	1.3	**<0.001**
Waist circumference (cm)	12	55.8	5.3	441	60.3	8.2	101	63.6	10.5	**<0.001**
Skinfolds (mm)	12	15.3^a^	8.2	444	19.7	11.0	101	20.1	11.9	0.02^a^
Blood parameters										
Total cholesterol (mg/dL)	12	161.2	31.4	438	167.0	27.2	100	167.5	24.7	0.82
HDL-cholesterol (mg/dL)	12	51.3	14.2	436	50.3	10.3	101	50.6	10.9	0.97
Triglycerides (mg/dL)	12	109.4	35.7	437	98.4	33.8	101	94.6	28.3	0.26
LDL-cholesterol (mg/dL)	12	88.0	25.0	435	97.2	24.6	100	97.6	23.0	0.59
Glycemia (mg/dL)	12	82.2	6.5	438	82.3	7.8	101	83.0	6.5	0.73
HOMA-IR	12	1.15^b^	0.8–1.5^c^	438	1.0^b^	0.6–1.5^ c^	101	0.98^ b^	0.7–1.4^ c^	0.03^a^
Insulin (*µ*/dL)	12	5.4^b^	4.5–7.1^c^	438	5.0^b^	3.2–7.2^c^	101	4.7^b^	3.4–6.7^ c^	0.02^a^
hs-CRP (mg/dL)	12	0.01^b^	0.01-0.02^c^	438	0.01^b^	0.01–0.06^c^	101	0.0^b^	0.01–0.09^c^	**<**0.001^a^

^a^Nonparametric test—General Linear Model—Gamma distribution.

^b^Median.

^c^P25–75.

SGA: small for gestational age.

AGA: appropriate for gestational age.

LGA: large for gestational age.

HDL: high density lipoprotein.

LDL: low density lipoprotein.

HOMA-IR: homeostasis model assessment method for insulin resistance.

hs-CRP: high sensitivity c reactive protein.

**Table 3:** Adjusted association between birth weight and anthropometric markers, lipid profile, glucose, HOMA-IR index, and hs-CRP in Brazilian school children (*n* = 612), 2013.

	SGA birth			AGA birth			LGA birth			*P* value
	*n*	Mean	SEM	*n*	Mean	SEM	*n*	Mean	SEM	
Anthropometric parameters^a^										
Body mass index (*z*-score)	12	−0.65	0.39	444	0.53	0.06	102	0.88	0.14	**<**0.001^c^
Waist circumference (cm)	12	56.0	2.5	441	60.3	0.41	101	63.4	0.86	0.001^c^
Skinfolds (mm)	12	14.6	3.4	444	19.7	0.55	101	22.7	1.17	0.002^d^
Blood parameters^b^										
Total cholesterol (mg/dL)	12	162.7	7.7	438	166.6	1.3	100	167.2	2.7	0.85^c^
HDL-cholesterol (mg/dL)	12	51.6	3.0	436	50.3	0.5	101	50.4	1.0	0.92^c^
Triglycerides (mg/dL)	12	112.1	8.8	437	98.4	1.4	101	93.7	3.0	0.10^c^
LDL-cholesterol (mg/dL)	12	88.7	6.9	435	96.8	1.15	100	97.9	2.4	0.45^c^
Glycemia (mg/dL)	12	82.6	2.2	438	83.1	0.4	101	82.4	0.8	0.72^c^
HOMA-IR	12	1.5	0.25	438	1.13	0.03	101	1.12	0.06	0.18^d^
Insulin (*µ*/dL)	12	7.5	1.18	438	5.4	0.14	101	5.5	0.31	0.14^d^
hs-CRP (mg/dL)	12	0.02	0.01	438	0.12	0.01	101	0.15	0.02	**<**0.001^d^

^a^Adjusted for gender.

^b^Adjusted for gender, body mass index, waist circumference, and skinfolds.

^c^General Linear Model—Normal Distribution.

^d^Nonparametric test—General Linear Model—Gamma distribution.

SEM: Standard Error Mean.

SGA: small for gestational age.

AGA: appropriate for gestational age.

LGA: large for gestational age.

HDL: high density lipoprotein.

LDL: low density lipoprotein.

HOMA-IR: homeostasis model assessment method for insulin resistance.

hs-CRP: high sensitivity c reactive protein.
